# Perchloric acid-soluble proteins from goat liver inhibit chemical carcinogenesis of Syrian hamster cheek-pouch carcinoma

**DOI:** 10.1038/sj.bjc.6690011

**Published:** 1999-09-24

**Authors:** F Ghezzo, G N Berta, B Bussolati, A Bosio, G Corvetti, F Di Carlo, G Bussolati, R Guglielmone, A Bartorelli

**Affiliations:** Department of Biological and Clinical Sciences, University of Turin, Turin, Italy; Department of Medical and Surgical Sciences, University of Turin, Turin, Italy; Department of Biomedical Sciences and Oncology, University of Turin, Turin, Italy; G Sisini Institute, University of Milan, Milan, Italy

**Keywords:** Calmette–Guérin bacillus, cytotoxicity, dexamethasone, hamster cheek-pouch carcinoma, Mib5, tumour inhibition; UK101

## Abstract

Chemically induced Syrian hamster cheek-pouch squamous cell carcinoma is very similar to the corresponding human tumour. This paper describes a blind study in which inhibition of dimethylbenzanthracene-induced cheek-pouch tumours by a goat liver extract denominated UK101 was investigated. Less than 40% of animals treated with UK101 developed tumours compared with 100% of the controls. Intermediate results (80%) were noted in a positive control group treated with Calmette–Guérin bacillus. Immunocytochemical testing of cheek-pouch mucosa by Mib5 showed significantly less proliferating cells in UK101 animals than in the controls. The effect of UK101 was completely reversed when dexamethasone was added in a third control group. A significant difference in complement-mediated cytotoxicity was noted in the sera of UK101-tested and control animals. These findings suggest that an immune mechanism is responsible for the inhibition of hamster cheek-pouch carcinoma by UK101. © 1999 Cancer Research Campaign


*British Journal of Cancer* (1999) **79**(1), 54–58
© 1999 Cancer Research Campaign

### Perchloric acid-soluble proteins from goat liver inhibit chemical carcinogenesis of Syrian hamster cheek-pouch carcinoma

**F Ghezzo[1], GN Berta[1], B Bussolati[2], A Bosio[1], G Corvetti[1], F Di Carlo[1], G Bussolati[3], R Guglielmone[1] and A Bartorelli[4]**

Departments of [1]Biological and Clinical Sciences, [2]Medical and Surgical Sciences, [3]Biomedical Sciences and Oncology, University of Turin, Italy; [4]G Sisini Institute, University of Milan, Italy

**Summary** Chemically induced Syrian hamster cheek-pouch squamous cell carcinoma is very similar to the corresponding human tumour. This paper describes a blind study in which inhibition of dimethylbenzanthracene-induced cheek-pouch tumours by a goat liver extract denominated UK101 was investigated. Less than 40% of animals treated with UK101 developed tumours compared with 100% of the controls. Intermediate results (80%) were noted in a positive control group treated with Calmette–Guérin bacillus. Immunocytochemical testing of cheek-pouch mucosa by Mib5 showed significantly less proliferating cells in UK101 animals than in the controls. The effect of UK101 was completely reversed when dexamethasone was added in a third control group. A significant difference in complement-mediated cytotoxicity was noted in the sera of UK101-tested and control animals. These findings suggest that an immune mechanism is responsible for the inhibition of hamster cheek-pouch carcinoma by UK101.

**Keywords:** Calmette–Guérin bacillus; cytotoxicity; dexamethasone; hamster cheek-pouch carcinoma; Mib5; tumour inhibition; UK101

Mammalian perchloric acid-soluble liver extracts (Lévy-Favatier et al, 1993; Bartorelli et al, 1994; Oka et al, 1995; Ceciliani et al, 1996*a*), as well as a trichloroacetic acid-soluble extract from human mononuclear monocytes (Schmiedeknecht et al, 1996), have been shown to contain a protein with an unidentified function that may participate in cell differentiation and degeneration through regulation of protein synthesis. Vivacious tumour expression of this new family of proteins suggests that it may be involved in immune control of tumour growth (Bartorelli A et al, 1996). UK101, a perchloric goat liver extract, contains three main electrophoretically distinguishable bands of about 8.5, 14 and 50 kDa. The first seems to be ubiquitin and the second a protein of the new family, whereas the third, a glycoprotein with mannose residues, has not yet been fully defined (Ceciliani et al, 1996*b*). Attention has been primarily directed to the 14-kDa band (denominated UK114). Its role in tumour cell proliferation, however, is still uncertain. Complement-mediated cytotoxic activity has been demonstrated in the sera of UK114-treated animals (Bartorelli A et al, 1996), and both cytolysis and tumour inhibition have been observed in the sera of cancer patients (Bussolati et al, 1997). The therapeutic effect of UK101 and UK114 in experimental mammalian tumours has been studied (Bartorelli et al, 1994; Racca et al, 1997), whereas their role in the prevention of carcinogenesis has not been investigated.

Oral cancer (ICD9 140–149) is the sixth most common neoplasm worldwide (Parkin et al, 1993). We have devoted our attention to growth inhibition of its experimental analogue in animals.

The Syrian hamster has been used in experimental studies of various mouth diseases. It has long been known (Salley, 1954) that squamous cell carcinomas can be produced in the cheek pouches of hamsters by multiple brushings with chemicals such as dimethylbenzanthracene (DMBA), and they have, therefore, been extensively considered in investigation of mucosal malignancies (Shklar, 1972). Use of the hamster model has led to the recognition of substances that can be used to prevent these tumours, such as retinoic acid (Gilmore and Giunta, 1981), vitamin E (Trickler and Shklar, 1987), extracts of *Spirulina–Dunaliella* algae (Schwartz et al, 1988), bacille bilié de Calmette–Guérin (BCG) (Giunta et al, 1974) and levamisole (Eisenberg and Shklar, 1977). A recent analysis of *p53* gene expression (Gimenez-Conti et al, 1996) has also shown that the hamster cheek-pouch model is better than the mouse skin two-phase carcinogenesis model (Odukoya and Shklar, 1982) in the investigation to study human oral carcinogenesis due to smoke, smokeless tobacco and alcohol abuse.

The present paper assesses the ability of UK101 to inhibit carcinogenesis of DMBA-induced Syrian hamster cheek-pouch squamous cell carcinoma. The positive control was provided by animals subjected to an active immune treatment (BCG). Counteraction of the effect of UK101 by the corticosteroid hormone dexamethasone (DM) was also investigated.

*Received 14 January 1998*
*Revised 14 May 1998*
*Accepted 17 June 1998*

*Correspondence to:* F Ghezzo, Dipartimento di Scienze, Cliniche e Biologiche, Università di Torino, Ospedale San Luigi Gonzaga, Regione Gonzole 10, 10043 Orbassano (TO), Italy

### MATERIALS AND METHODS

### Animals and treatment

Animals were treated in accordance with the Italian legislation on animal experiments and under strict veterinary control in keeping with the UKCCCR guidelines.

Thirty-six 30- to 40-day-old male Syrian hamsters weighing about 50 g were randomly divided into five groups: control [eight animals treated with phosphate-buffered saline (PBS)], BCG (Pasteur-Mérieux, France) (eight treated with $1\times10^3$ culturable BCG particles diluted in PBS), UK101 (Sicor, Italy) (eight treated with 20 μg UK101 diluted in PBS), UK101 + DM (eight treated with 20 μg UK101 diluted in PBS plus 10 μg DM diluted in PBS, not mixed in the same syringe) and DM (four treated with 10 μg DM). The first three groups were blind treated. Each animal received 1 ml volume once a week subcutaneously during the whole course of the experiment, except for DM (twice a week owing to its biological half-life) (Schimmer and Parker, 1996). After 4 weeks from the beginning of subcutaneous administrations, the right cheek pouch of fasting animals was painted three times a week with 0.5% DMBA (Sigma, Italy) in paraffin oil. Animals were inspected for the presence of tumours once a week. After 18 weeks from the beginning of tumour induction, when frank tumours were evident in 100% of one of the first blind groups, the animals were killed by carotid bleeding under carbon dioxide anaesthesia. Their right cheek pouches were macroscopically examined to count and measure exophytic tumours and remove them for histological examination.

Tumours were grouped according to their major diameter as less or more than 2 mm (limit of macroscopic examination). The left pouches were also searched for tumours and none were found. The animals were then dissected for macroscopic examination of their principal organs. These were free from evident abnormalities. Although by macro- and microscopic examination no endophytic lesions were observed in the UK-treated animals, whereas endophytic lesions in the controls were always associated with the exophytic ones in the same area, the simplest, most objective and evident way of considering exophytic lesions only was chosen.

### Histology and immunocytochemical staining of proliferating cells

Specimens of tumours and left pouch mucosa were fixed in Carnoy's fluid or in formalin at 4°C for 12 h and embedded in paraffin. Sections (4 μm thick) were stained with haematoxylin and eosin. For immunocytochemistry, histologically normal areas were selected from the DMBA-treated mucosa of both controls and UK101-treated animals. Sections were stained with the Mib-5 monoclonal antibody (Immunotech, France) which reacts with the proliferation-associated Ki-67 antigen (Schluter et al, 1993). The antibody was diluted 1:10 in PBS and used after microwave treatment in citrate buffer. Nuclei were counterstained with haemalum. The thickness of the epithelium was similar in the two groups and ranged from 45 to 85 μm (mean 65 μm). Positive nuclei were counted in the basal and Malpighian layers over a length of epithelium of 250 μm from several sections and cases. Thirty-two specimens were obtained from three control animals and 56 obtained from seven UK101 animals.

### Electrophoresis

Standard techniques were employed for sodium dodecyl sulphate polyacrylamide gel electrophoresis (SDS-PAGE) and Western blot analysis (Sambrook et al, 1989) by using the enhanced chemiluminescence (ECL) detection system (Amersham, UK): rabbit polyclonal antibodies, 1:200 (Bartorelli et al, 1996), and commercial mouse monoclonal antibodies (Chemicon International, USA) were used to stain UK101 and ubiquitin, respectively, the former being recognized by horseradish peroxidase-protein A (Biorad, USA) and the latter by horseradish peroxidase-sheep anti-mouse (Amersham). Moreover, sera from UK-treated animals, 1:50, recognized by horseradish peroxidase–protein A, were also used to stain UK101 proteins. Ubiquitin was obtained from bovine red blood cells (Sigma).

### Cytotoxicity

Hamster sera were collected, decomplemented by heating for 1 h at 56°C and stored at –20°C until use. Human gastric cancer cells Kato III, expressing UK114 protein on their surface, and T47D, negative for UK114 expression, were labelled with 3.7 MBq of [$^{51}$Cr]sodium chromate for 1 h at 37°C and washed to remove extracellular $^{51}$Cr. Aliquots of the labelled cells were placed in 96-well microplates ($5\times10^4$ per 50 μl) and incubated for 5 h at 37°C with 50 μl of human serum at different concentrations in the presence of 10 μl of guinea pig complement (Sigma). Hyperimmune rabbit serum raised against UK114 protein was used as positive control. To evaluate the complement dependency of the reaction, control experiments were performed in the absence of complement. After centrifugation, release of $^{51}$Cr in the supernatant was counted. The percentage of specific cytolysis was calculated from the count of experimental release, total release and spontaneous release (Bartorelli et al, 1996).

### Statistics

Analysis of variance was used (Armitage, 1971).

### RESULTS

Photographs of right internal mucosa with cheek-pouch tumours in the control, BCG, UK101 + DM, and UK101 groups are presented in Figure 1. Microphotographs of examples from the control and treated groups (Figure 2) show that the control displays features of epidermoid carcinoma, very similar to those of the UK101 + DM group, with the exception of abundant stromal tissue in the latter,

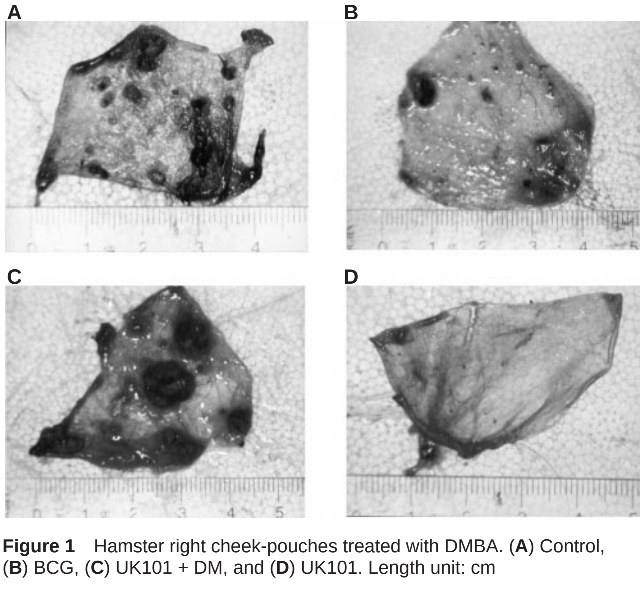

**Figure 1** Hamster right cheek-pouches treated with DMBA. (**A**) Control, (**B**) BCG, (**C**) UK101 + DM, and (**D**) UK101. Length unit: cm

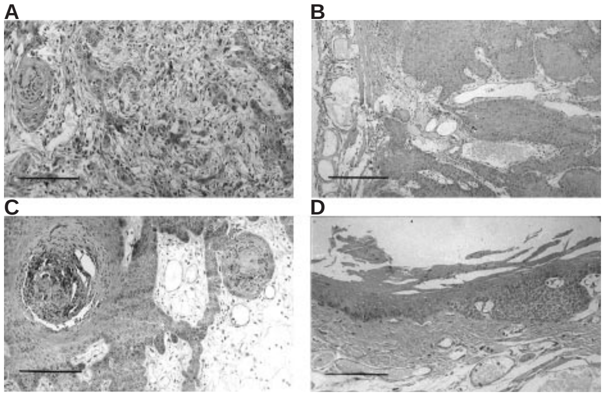

**Figure 2** Histological examination of hamster cheek-pouch tumours. (**A**) Control, bar=125 μm; (**B**) BCG, bar=200 μm; (**C**) UK101 + DM, bar=200 μm; (**D**) UK101, bar=200 μm

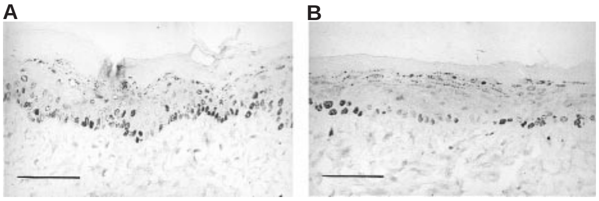

**Figure 3** Immunochemical staining of proliferating cells of histologically normal mucosa. (**A**) Control animals. (**B**) UK101-treated animals. Bar=80 μm

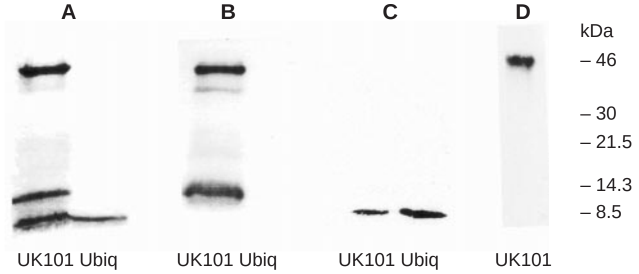

**Figure 4** (**A**) SDS-PAGE of the UK101 mixture and of ubiquitin. (**B**) Western blot analysis of UK101 and ubiquitin stained with anti-UK101 rabbit polyclonal antibodies. (**C**) Western blot analysis of UK101 and ubiquitin stained with mouse monoclonal antibodies against ubiquitin. (**D**) Western blot analysis of UK101 stained by UK101-treated hamster serum whereas BCG tumour shows less marked signs of malignancy and that of the UK101 group was the least malignant. Tumours of the DM group, not reported in the figures, were indistinguishable from control. Table 1 summarizes the characteristics of these specimens.

Inhibition of the tumour growth in the UK101-treated animals could be related to a lower proliferation of the epithelial layer. To test this hypothesis, we established an immunocytochemical procedure using the Mib5 antibody revealing the proliferation-associated Ki-67 nuclear antigen (Figure 3). Values obtained in histologically normal areas of the DMBA-treated mucosa of both controls and UK101-treated tests were compared. Values were significantly different ($P < 0.0001$) in controls (mean 22.8 ± 5.3) and in UK101-treated animals (10.7 ± 2.8). In Figure 4, SDS-PAGE and Western blotting of the UK101 preparation together with ubiquitin are shown. As expected, UK101 SDS-PAGE shows three main bands of 50, 14 and 8.5 kDa respectively. The last molecular weight was coincident with that of ubiquitin. Western blotting analysis with rabbit anti-UK101 antibodies shows clearly the two main bands of the SDS-PAGE at 14 and 50 kDa, together with a fainter band at about 30 kDa. The absence of the band evident at 8.5 kDa in the SDS-PAGE is probably indicative of the poor antigenic power of the small ubiquitin molecule. A Western blot with mouse monoclonal antibodies against ubiquitin shows the identity of the third band with ubiquitin. In the last lane of the same table, an example of UK101 stained by serum of a UK-treated animal is also reported. The presence of only one band could be evidence of the poorly antigenic power of the UK114 fraction when Freund's adjuvant is not employed.

The tumour numbers and sizes in the five groups are listed in Table 2. Both UK101-treated and, to a significantly lesser extent, BCG-treated animals showed fewer tumours than the controls. The UK101+DM and DM groups resembled the controls. The incidence of the small (<2 mm) and the large (>2 mm) tumours was statistically similar.

Table 3 shows the results of the cytotoxicity experiments. Sera from the UK101 group displayed greater cytotoxicity than sera from the controls and BCG when UK114-positive Kato III cells were used. The cytotoxic effect was absent when UK114-negative T47D cells were used (data not shown). DM seemed to completely reverse the effect of UK101.

### DISCUSSION

As observed by other authors, DMBA painting of hamster cheek pouches produced tumours very similar to human squamous cell carcinoma of the oral cavity, and BCG was able to oppose this carcinogenetic mechanism. UK101 was stronger than BCG in inhibiting carcinogenesis. The immunocytochemical data also

**Table 1** Microscopic characteristics of right cheek-pouch carcinomas

| Group | Stroma | Vessels | Horn pearl | Hyalinosis | Nuclear atypia | Local malignancy |
|---|---|---|---|---|---|---|
| Control and DM | + | ++ | ++/+++ | + | ++/+++ | + |
| BCG | + | + | ++ | + | + | –/+ |
| UK101 | + | –/+ | –/+ | –/+ | –/+ | – |
| UK101 + DM | ++/+++ | ++ | ++/+ | + | +++/++ | –/+ |

–, Absent; +, barely present; ++, present; +++, abundant.

 *© Cancer Research Campaign 1999*

**Table 2** Effect of UK101 in inhibiting DMBA-induced hamster cheek-pouch carcinoma

| | < 2 mm | >2 mm | Total |
|---|---|---|---|
| Control | 9.2 ± 5.8 | 3.0 ± 2.2 | 12.2 ± 5.6 |
| BCG | 3.2 ± 2.8 | 2.6 ± 1.9 | 5.9 ± 3.6 |
| vs control | * | * | ** |
| UK101 | 0.5 ± 1 | 0.4 ± 0.7 | 0.9 ± 1.2 |
| vs control | **** | **** | **** |
| vs BCG | * | * | **** |
| vs UK101 + DM | **** | **** | **** |
| vs DM | **** | **** | **** |
| UK101 + DM | 5.7 ± 3.9 | 4.3 ± 2.8 | 10.0 ± 5.2 |
| vs control | ns | ns | ns |
| vs DM | ns | ns | ns |
| DM | 7.2 ± 3.5 | 4.7 ± 2.9 | 12 ± 5.9 |
| vs control | ns | ns | ns |

ns, Not significant; *$P$<0.05; **$P$<0.025; ***$P$<0.01; ****$P$<0.005.

**Table 3** Enhancement of complement-mediated cytotoxicity by UK101

| Groups | Cytotoxicity (% cell killing) |
|---|---|
| Control | 12.4 ± 4.8 |
| BCG | 12.3 ± 4.3 |
| vs control | ns |
| UK101 | 19.6 ± 2.7 |
| vs control | * |
| vs BCG | ** |
| vs UK101 + DM | ** |
| UK101 + DM | 13.7 ± 2.7 |
| vs control | ns |

ns, Not significant; *$P$<0.05; **$P$<0.025.

show active inhibition of proliferation of the epithelial layer by UK101 when tumour lesions are not yet evident, suggesting the presence of a mechanism of action able to act in the early phases of carcinogenesis. However, because no particular difference could be observed in the incidence of small and large tumours, it may be suggested that UK101 is also effective in the late stages of tumour growth. A direct effect of UK101 on the activation and alkylating mechanisms of DMBA cannot be ruled out. Although corticosteroids have many types of actions, the observation that DM reversed the effect of UK101, as shown by the absence of a significant difference between the control and the UK101+DM groups, may reflect the result of an immunosuppressive action of the drug, suggesting involvement of the immune system in UK101 antitumour activity. Elucidation of the precise mechanism of action of glucocorticoids is beyond the purpose of this work. Even so, DM opposition to anti-tumour activity against the product of the action of DMBA rather than against the action of DMBA itself seems the more reasonable hypothesis. In either event, the phenomenon lends support to the reflection that the use of corticosteroids may not be indifferent to the efficacy of the antitumoral therapy. The cytotoxicity data are further evidence in favour of a stimulus of serum anti-tumour antibody production as a possible mechanism of the action of UK101. Here, too, DM abolished UK101-stimulated cytotoxicity.

Assuming that UK101 activates the immune system against tumours, Western blot analysis showing that it stimulates production of antibodies directed against principally the 14- and 50-kDa bands, and to a lesser extent the 37-kDa band, suggests that they can constitute the most important antigenic stimulus and are capable of unleashing an anti-tumour reaction. This may be true even if our subcutaneous administration without Freund's adjuvant cannot be simply superimposed on classic immunological techniques for producing antibodies like those used to produce our rabbit antiserum. In fact, only the 50-kDa band is shown when serum of UK-treated hamsters is used to stain UK101. This may simply reflect the greater efficacy of higher molecular weight protein in stimulating antibody production revealable by Western blot analysis. It does, however, urgently require further work to clarify the role of each UK101 fraction in stimulating the anti-tumour immune reaction.

Our opinion is that other studies are necessary to reveal the exact mechanism of action of the perchloric extract UK101, determine which of its fractions is active, and demonstrate or rule out their cooperation. Nonetheless, our data provide clear evidence that it inhibits chemically induced carcinogenesis in the Syrian hamster and stimulates serum-mediated cytotoxicity against tumour cells such as Kato III.

### ACKNOWLEDGEMENTS

This work was supported by the University of Turin and the company Sicor (Rho, Milan, Italy). We are also indebted to J Iliffe and D Concas-Benevelli for helpful discussion.

### REFERENCES

Armitage P (1971) *Statistical Methods in Medical Research*. Blackwell: Scientific Publications: Oxford

Bartorelli A, Zanolo G, Fassio F, Botta M, Ferrara R, Bailo M, Biancardi C, Cavalca V, Arzani C and Maraschin R (1994) Toxicological and antitumoral activity of UK101, a mammalian liver extract. *J Tumor Marker Oncol* **9**: 49–56

Bartorelli A, Bussolati B, Millesimo M, Gugliotta P and Bussolati G (1996) Antibody-dependent cytotoxic activity on human cancer cells expressing UK114 tumor membrane antigen. *Int J Oncol* **8**: 543–548

Bussolati G, Geuna M, Bussolati B, Millesimo M, Botta M and Bartorelli A (1997) Cytolytic and tumor inhibitory antibodies against UK114 protein in the sera of cancer patients. *Int J Oncol* **10**: 779–785

Ceciliani F, Faotto L, Negri A, Colombo I, Berra B, Bartorelli A and Ronchi S (1996a) The primary structure of UK114 tumor antigen. *FEBS Lett* **393**: 147–150

Ceciliani F, Biancardi C, Cavalca V, Ferrara R, Botta M, Arzani C, Bailo M, Berra B, Ronchi S and Bartorelli A (1996b) Structural characterization of the small molecular weight proteins present in UK101. *J Tumor Marker Oncol* **11**: 63–66

Eisenberg E and Shklar G (1977) Levamisole and hamster pouch carcinogenesis. *Oral Surg Oral Med Oral Pathol* **43**: 562–571

Gilmore W and Giunta J (1981) The effect of 13-*cis*-retinoic acid on hamster buccal pouch carcinogenesis. *Oral Surg Oral Med Oral Pathol* **51**: 256–264

Gimenez-Conti IB, LaBate M, Liu F and Osterndorff E (1996) p53 alterations in chemically induced hamster cheek-pouch lesions. *Mol Carcinog* **16**: 197–202

Giunta JL, Reif AE and Shklar G (1974) Bacillus Calmette-Guérin and antilymphocyte serum in carcinogenesis. *Arch Pathol* **98**: 237–240

Lévy-Favatier F, Cuisset L, Nedelec B, Tichonicky L, Kruh J and Delpech M (1993) Characterization, purification and cDNA cloning of a rat perchloric-acid-soluble 23-kDa protein present only in liver and kidney. *Eur J Biochem* **212**: 665–673

Odukoya O and Shklar G (1982) Two-phase carcinogenesis in hamster buccal pouch. *Oral Surg Oral Med Oral Pathol* **54**: 547–552

Oka T, Tsuji H, Noda C, Sakai K, Hong Y, Suzuki I, Munoz S and Natori Y (1995) Isolation and characterization of a novel perchloric acid-soluble protein inhibiting cell-free protein synthesis. *J Biol Chem* **270**: 30060–30067

Parkin DM, Pisani P and Ferlay J (1993) Estimates of the worldwide incidence of eighteen major cancers in 1985. *Int J Cancer* **54**: 594–606

Racca S, Di Carlo F, Bartorelli A, Bussolati B and Bussolati G (1997) Growth inhibition of DMBA-induced rat mammary carcinomas by UK114. *Virchow Arch* **431**: 323–328

Salley JJ (1954) Experimental carcinogenesis in the cheek pouch of the Syrian hamster. *J Dent Res* **33**: 253–262

Sambrook J, Fritsch EF and Maniatis T (1989) *Molecular Cloning*. Cold Spring Harbor Laboratory Press: Cold Spring Harbor

Schimmer BP and Parker KL (1996) Adrenocorticotropic hormone; adrenocortical steroids and their synthetic analogs; inhibitors of the synthesis and actions of adrenocortical hormones. In *The Pharmacological Basis of Therapeutics*. Hardman JG and Limbird LE (eds), pp. 1459–1485. McGraw-Hill: New York

Schluter C, Durchrow M, Wohlenberg C, Becker MHG, Key G, Flad HD and Gerdes J (1993) The cell proliferation-associated antigen of antibody Ki-67: a very large, ubiquitous nuclear protein with numerous repeated elements, representing a new kind of cell cycle maintaining protein. *J Cell Biol* **123**: 513–522

Schmiedeknecht G, Kerkhoff C, Orso E, Stohr J, Aslandis C, Nagy GM, Knuechel R and Schmitz G (1996) Isolation and characterization of a 14.5-kDa trichloroacetic-acid-soluble translational inhibitor protein from human monocytes that is upregulated upon cellular differentiation. *Eur J Biochem* **242**: 339–351

Schwartz J, Shklar G, Reid S and Trickler D (1988) Prevention of experimental oral cancer by extracts of Spirulina-Dunaliella algae. *Nutr Cancer* **11**: 127–134

Shklar G (1972) Experimental oral pathology in the Syrian hamster. *Prog Exp Tumor Res* **16**: 518–538

Trickler D and Shklar G (1987) Prevention by vitamin E of experimental oral carcinogenesis. *J Natl Cancer Inst* **78**: 165–169

*© Cancer Research Campaign 1999*

## References

[PDF_00356] Andronico G., Mangano M., Ferrara L., Lamanna D., Mulé G., Cerasola G. (1997). In vivo relationship between insulin and endothelin role of insulin-resistance.. J Hum Hypertens.

[PDF_00353] Ceciliani F., Faotto L., Negri A., Colombo I., Berra B., Bartorelli A., Ronchi S. (1996). The primary structure of UK114 tumor antigen.. FEBS Lett.

[PDF_00360] Eisenberg E., Shklar G. (1977). Levamisole and hamster pouch carcinogenesis.. Oral Surg Oral Med Oral Pathol.

[PDF_00362] Gilmore W., Giunta J. L. (1981). The effect of 13-cis-retinoic acid on hamster buccal pouch carcinogenesis.. Oral Surg Oral Med Oral Pathol.

[PDF_00364] Gimenez-Conti I. B., LaBate M., Liu F., Osterndorff E. (1996). p53 alterations in chemically induced hamster cheek-pouch lesions.. Mol Carcinog.

[PDF_00367] Giunta J. L., Reif A. E., Shklar G. (1974). Bacillus Calmette-Guérin and antilymphocyte serum in carcinogenesis. Effects on the hamster pouch.. Arch Pathol.

[PDF_00369] Levy-Favatier F., Cuisset L., Nedelec B., Tichonicky L., Kruh J., Delpech M. (1993). Characterization, purification and cDNA cloning of a rat perchloric-acid-soluble 23-kDa protein present only in liver and kidney.. Eur J Biochem.

[PDF_00373] Odukoya O., Shklar G. (1982). Two-phase carcinogenesis in hamster buccal pouch.. Oral Surg Oral Med Oral Pathol.

[PDF_00375] Oka T., Tsuji H., Noda C., Sakai K., Hong Y. M., Suzuki I., Muñoz S., Natori Y. (1995). Isolation and characterization of a novel perchloric acid-soluble protein inhibiting cell-free protein synthesis.. J Biol Chem.

[PDF_00378] Parkin D. M., Pisani P., Ferlay J. (1993). Estimates of the worldwide incidence of eighteen major cancers in 1985.. Int J Cancer.

[PDF_00380] Racca S., Di Carlo F., Bartorelli A., Bussolati B., Bussolati G. (1997). Growth inhibition of DMBA-induced rat mammary carcinomas by UK 114.. Virchows Arch.

[PDF_00383] SALLEY J. J. (1954). Experimental carcinogenesis in the cheek pouch of the Syrian hamster.. J Dent Res.

[PDF_00391] Schlüter C., Duchrow M., Wohlenberg C., Becker M. H., Key G., Flad H. D., Gerdes J. (1993). The cell proliferation-associated antigen of antibody Ki-67: a very large, ubiquitous nuclear protein with numerous repeated elements, representing a new kind of cell cycle-maintaining proteins.. J Cell Biol.

[PDF_00396] Schmiedeknecht G., Kerkhoff C., Orsó E., Stöhr J., Aslanidis C., Nagy G. M., Knuechel R., Schmitz G. (1996). Isolation and characterization of a 14.5-kDa trichloroacetic-acid-soluble translational inhibitor protein from human monocytes that is upregulated upon cellular differentiation.. Eur J Biochem.

[PDF_00401] Schwartz J., Shklar G., Reid S., Trickler D. (1988). Prevention of experimental oral cancer by extracts of Spirulina-Dunaliella algae.. Nutr Cancer.

[PDF_00403] Shklar G. (1972). Experimental oral pathology in the Syrian hamster.. Prog Exp Tumor Res.

[PDF_00405] Trickler D., Shklar G. (1987). Prevention by vitamin E of experimental oral carcinogenesis.. J Natl Cancer Inst.

